# Adhesion awareness in 2016: An update of the national survey of surgeons

**DOI:** 10.1371/journal.pone.0202418

**Published:** 2018-08-17

**Authors:** Sebastiaan van Steensel, Leontine C. L. van den Hil, Marc H. F. Schreinemacher, Richard P. G. ten Broek, Harry van Goor, Nicole D. Bouvy

**Affiliations:** 1 Department of General Surgery, Maastricht University Medical Centre, Maastricht, The Netherlands; 2 NUTRIM School of Nutrition and Translational Research in Metabolism, Maastricht University, Maastricht, The Netherlands; 3 Department of General Surgery, Academic Medical Centre, Amsterdam, The Netherlands; 4 Department of General Surgery, Radboud University Medical Centre, Nijmegen, The Netherlands; Campus Bio Medico University, ITALY

## Abstract

**Background and aims:**

Adhesions, that form in 60–80% of all abdominal operations, can cause complications such as chronic abdominal pain, small-bowel obstruction, female infertility, and the need for adhesiolysis in future surgeries. Our 2010 Adhesion Awareness survey demonstrated that despite the huge clinical impact of adhesions; adhesion-related complications were seldom mentioned in the informed consent. Six years later, a follow-up survey was conducted to assess the progress on awareness on adhesion-related complications in the Netherlands.

**Material and methods:**

The 2010 Adhesion Awareness survey was repeated after a literature update. The knowledge regarding adhesions; the use of anti-adhesive agents and involvement in the informed consent process were assessed. Surgeons and surgical trainees were contacted by e-mail. The data was analysed using a Chi-square or Mann-Whitney U test and corrected for multiple testing.

**Results:**

The response rate was 32.6%, similar to the survey in 2010 (34.4%). 88.1% agreed with the clinical relevance of adhesions, comparable to 2010 (89.8%). The score on the knowledge test was 38.8% (2010: 37.2%). Involvement of adhesion-related complications in the informed consent process increased, although 32.5% almost never mentions adhesions. In 2016, 42.4% reported a correct occurrence of bowel lesions during adhesiolysis, higher than in 2010 (P<0.001).

**Conclusions:**

The adhesion awareness did not increase in six years, despite the efforts made. However, an increased awareness regarding adhesiolysis related complications was detected. Improvement of knowledge and behavior is essential to narrowing the gap between the impact of adhesions as a major complication of abdominal surgery and the limited adhesion awareness.

## Introduction

Abdominal surgery leads to intraperitoneal adhesion formation in almost all patients (60–80%), often giving rise to complications in the short, but also in the medium and long-term [1, 2]. Adhesion related complications comprise of chronic abdominal pain, small-bowel obstruction and female infertility [3]. Adhesions are responsible for 56% of the postoperative small-bowel obstruction, are the most likely cause of chronic abdominal pain after a former laparotomy in 57% of the patients, and effects 23% of the female patients who require fertility treatment after abdominal surgery [3]. Additional to the direct complications of adhesions there is also an increased risk of adverse events during future surgery because of the need for adhesiolysis accompanied with increased risk of bowel injury[3].

The direct link between abdominal surgery and adhesion-related complications is often overlooked because of the long period between the occurrence of complications and the initial surgery [3]. However, the clinical burden is extensive considering that up to a third of patients that have undergone abdominal surgery will be readmitted for adhesion related complications in the next 10 years. Moreover, a mean readmission rate of 2.2 per patient has previously been reported [4, 5]. Thus, adhesions must be regarded as one of the most important complications after abdominal surgery.

However, there is a discrepancy between the clinical impact of adhesion related morbidity and the provided information regarding this topic during the informed consent process preoperatively [6]. Therefore, it was hypothesized that surgeons lack sufficient awareness of this common complication and its possible impact. To investigate this, a survey among surgeons was performed in 2010 in the Netherlands [7]. This survey showed that two out of three Dutch surgeons recognized adhesions as a serious problem, but the readmission rates and the incidence of small bowel obstructions caused by adhesions were grossly underestimated. In response, a number of high quality reviews and meta-analyses demonstrating the impact of adhesions were published [3, 8, 9]. Results from these studies were presented during symposia and at national surgical meetings in order to raise awareness and educate about the problem and its possible prevention.

Six years after conducting the National Adhesions Awareness Survey we re-evaluated the adhesion awareness among surgeons in the Netherlands. A second National survey was conducted recently using the same questionnaire, with an update of the knowledge test using recent literature.

## Methods

The original survey in 2010 was conceived by the Dutch Adhesion Group, a steering group of 11 general and gynaecologic surgeons with affiliation to adhesion related morbidity. In order to provide comparable data, the survey of 2010 was used after a minor update. The answers of the knowledge test were adjusted to the most recent literature and some additional questions were added to evaluate the progression of adhesion awareness over time [7]. Only an online version was available, which was in Dutch and consisted of 62 multiple choice and five open questions with a total word count of 827 words. The complete translated survey can be found in the appendix (S1 Text). The surveys were filled out anonymously.

### Knowledge test

Ten multiple choice questions regarding prevalence and morbidity of adhesions were formulated to obtain information on the average know-how of participating surgeons. The following statements were considered correct:

- 56% of the small bowel-obstructions are caused by postoperative adhesions [3].
- The 5-years readmission rate after laparotomy directly related to postoperative adhesions is approximately 5% [10].
- The 10-years readmission rate after laparotomy probably related to postoperative adhesions is approximately 30% [4].
- Iatrogenic bowel injury occurs in 40% of the operations needing adhesiolysis of approximately one hour [3].
- The highest risk on adhesion formation is caused by a total colectomy in comparison to a partial small-bowel resection, resection of the rectum, appendectomy or cholecystectomy [10].
- Of the women that have a history of abdominal surgery, 23% will be treated for infertility [3, 11].
- An age above 60 years is associated with fewer adhesion formation compared to younger patients. A history of abdominal surgery is associated with more adhesion formation compared to patients with no prior abdominal surgery and Crohn’s disease has no effect on adhesion formation [10].

### Survey distribution

The questionnaire was distributed among 1582 surgeons and trainees using an online survey service (SurveyMonkey Inc, Palo Alto, California, USA; www.survey-monkey.com). All e-mail addresses were collected using the website of the Dutch Association for Surgery. The survey has been sent to all surgeons or trainees on a Tuesday (7:00am) and four reminders were sent afterwards, respectively one, two and three weeks after the initial e-mail, alternating at 7:00pm and at 7:00am. The last reminder was sent four weeks after the first invitation at 7.00am. One week after the last reminder the online survey was closed.

### Data analysis

Data was exported from the online survey service into IBM SPSS statistics, version 23.0 (SPSS, Inc., Chicago, Illinois, USA) for further analysis. Only surveys that were more than 80% completed were included for analysis. Results were compared to the 2010 survey. In case of normal distribution tested using the Shapiro-Wilk test, proportions were compared using the Pearson Chi-square test and for the comparison of means an independent student t-test was used. The Mann-Whitney U test was used when the data was not normally distributed. To take multiple testing into account, a Bonferroni corrected *P*-value of <0.0017 (0.05/29) was considered as statistically significant.

## Results

Of the 1582 surgeons and trainees contacted, a total response rate of n = 514 (32.5%) was generated, which was comparable with 2010 (34.4%). From the respondents, 25.7% also responded to the 2010 survey. 93 participants explicitly refused to participate and 41 surveys were excluded because they were incomplete. A total of 380 surveys (90.3%) was available for analysis. The group of respondents was divided in trainees (20.8%), general and gastro-intestinal surgeons combined (49.5%) and others (29.7%). Further demographic details are shown in Table 1.

**Table 1:** Baseline characteristics participants.

	2010	2016	*P*-value^a^
Male (%)	-	80.0	-
Trainees (%)	26.1	20.8	0.10
General/ GI surgeon (%)	49.1	49.5	0.10
Other type of surgeon (%)	24.8	29.7	0.10
Working experience (mean in years)	10.8	12.7	0.01
Non-academic hospital (%)	72.1	71.3	0.79
Fulltime (%)	89.1	83.7	0.02

Baseline characteristics expressed in percentages and means.

^a^ p values <0.05 were considered significant.

### Opinion on adhesions

Of all respondents, 88.1% agreed with the clinical relevance of adhesions, which is comparable to the 89.8% of the respondents agreeing with this statement in the survey of 2010 (*p* = 0.41). All outcomes are presented in Table 2. No significant differences were detected between trainees, gastrointestinal and general surgeons or other surgeons. 70.8% of the respondents agreed that adhesiolysis for diffuse abdominal complaints is an obsolete intervention. Regarding focal abdominal complaints only 27.2% agreed that adhesiolysis is senseless.

**Table 2:** Overview of the outcomes of the Adhesion Awareness survey; 2010 vs 2016.

Topic	2010 (n = 501)	2016 (n = 380)	*P*-value ^a^
Adhesions are not of clinical interest			0.41
Disagree	89.8%	88.1%	
Neutral	4.4%	5.5%	
Agree	5.8%	6.3%	
Adhesions have more beneficial than detrimental effects			0.005
Disagree	72.3%	63.4%	
Neutral	20.4%	27.1%	
Agree	7.2%	9.5%	
Adhesiolysis for diffuse abdominal pain complaints is not effective			-
Disagree	-	11.6%	
Neutral	-	17.6%	
Agree	-	70.8%	
Adhesiolysis for focal abdominal pain complaints is not effective			-
Disagree	-	47.5%	
Neutral	-	25.3%	
Agree	-	27.2%	
Adhesioloysis should preferably be performed by specialized gastrointestinal surgeons			-
Disagree	-	30%	
Neutral	-	15.8%	
Agree	-	54.2%	
Which proportion of small bowel obstruction is caused by postoperative adhesions?			0.002
Correct answer (%)	32.1%	42.4%	
Which percentage of patients will be readmitted within 5 years after laparotomy, due to morbidity directly related to adhesions?			0.40
Correct answer (%)	42.8%	40%	
Which percentage of patients will be readmitted within 10 years after laparotomy, due to morbidity probably related to adhesions?			0.01
Correct answer (%)	6.9%	11.8%	
What is the percentage of occurence of inadvertent bowel lesions caused by adhesiolysis?			<0.001*
Correct answer (%)	23.9%	42.4%	
Which procedure carries the highest risk for adhesion-related morbidity?			-
Correct answer (%)	-	70.8%	
Which percentage of the women with a history of abdominal surgery has to be treated for infertility?			-
Correct answer (%)	-	6.1%	
What is the influence of age >60years on adhesion formation?			0.50
Correct answer (%)	31.8%	33.9%	
What is the influence of a history of abdominal surgery on adhesion formation?			0.97
Correct answer (%)	87.6%	87.6%	
What is the influence of Crohn’s disease on adhesion formation?			0.39
Correct answer (%)	16.1%	13.9%	
How many patients do you inform about adhesion-related morbidity as a possible complication after laparotomy?			0.003
Virtually none	41.1%	32.5%	
<5%	15.2%	17.3%	
5–10%	11.2%	10.1%	
10–25%	7.6%	6.4%	
25–50%	6.4%	6.7%	
50–75%	3.0%	5.6%	
Virtually all	15.4%	21.3%	
How many patients do you inform about adhesion-related morbidity as a possible complication after laparoscopy?			0.60
Virtually none	64.3%	65.9%	
<5%	11.6%	10.4%	
5–10%	7.0%	6.9%	
10–25%	3.0%	2.9%	
25–50%	2.8%	4.8%	
50–75%	1.0%	2.7%	
Virtually all	10.2%	6.4%	
You do not believe in adhesion prevention			0.743
Disagree	39.1%	34.4%	
Neutral	38.5%	35.8%	
Agree	22.4%	29.8%	
You would like to apply adhesion prevention in all abdominal operations			<0.001*
Disagree	33.6%	45.8%	
Neutral	35.2%	25.3%	
Agree	31.2%	28.9%	
You would like to apply adhesion prevention only in certain indications			0.86
Disagree	19.2%	17.5%	
Neutral	27.7%	26.9%	
Agree	53.1%	55.7%	
Laparoscopic surgery causes fewer adhesions than open surgery			<0.001*
Disagree	23.4%	4.9%	
Neutral	0%	9.5%	
Agree	76.6%	85.6%	
Meticulous surgical technique reduces adhesions			<0.001*
Disagree	21.2%	8.2%	
Neutral	0%	16.3%	
Agree	78.8%	75.5%	
Extraperitoneal mesh position causes fewer adhesions than intraperitoneal mesh position			0.95
Disagree	4.6%	3.5%	
Neutral	8.8%	7.6%	
Agree	86.6%	88.8%	
A coated mesh causes fewer adhesions than an uncoated mesh			0.40
Disagree	5.2%	7.0%	
Neutral	26.4%	24.9%	
Agree	68.4%	68.0%	
Electrocautery causes fewer adhesions			-
Disagree	-	32.8%	
Neutral	-	56.1%	
Agree	-	11.1%	
Less intraperitoneal suture material reduces adhesions			0.32
Disagree	13.1%	9.8%	
Neutral	39.6%	33.2%	
Agree	47.3%	57.1	
You do not believe in antiadhesive agents			0.92
Disagree	26.5%	24.2%	
Neutral	44.3%	42.0%	
Agree	29.1%	33.8%	
You experience a lack of clarity about when to use an antiadhesive agents			0.005
Disagree	11.0%	6.3%	
Neutral	22.3%	28.6%	
Agree	66.7%	65.1%	
You prefer using a locally acting antiadhesive agent over an agent that acts throughout the whole abdomen			0.06
Disagree	31.3%	24.5%	
Neutral	50.3%	55.2%	
Agree	18.4%	20.3%	
You think the costs do not outweigh the possible benefits of antiadhesive agents			0.58
Disagree	9.2%	8.2%	
Neutral	57.4%	59.1%	
Agree	33.3%	32.7%	

^a^ a Bonferroni corrected *P*-value of <0.0017 (0.05/29) is considered significant.

* p value is considered significant

### Prevalence and morbidity of adhesions

The mean percentage of correct answers of the correspondents on the knowledge test was 38.8% (SD 13.0), not significantly different from the score in 2010 (37.2%, SD 15.2) (*p* = 0.92).

In 2016 42.4% reported a correct occurrence of iatrogenic bowel lesions during adhesiolysis, were in 2010 this was only 23.9% (p<0.001). The relation between the 5-years readmission rate after laparotomy and postoperative adhesions was reported correctly by 40.0%, whereas 10-years readmission rates were only reported correctly by 11.8% of the respondents. In both cases, no significant difference from the 2010 survey was detected. Of all participants, 70.8% reported correctly a total colectomy as surgery with the highest risk on adhesion formation. In contrast, 93.4% underestimated the necessity of fertility treatment in women that have a history of abdominal surgery. Furthermore, 33.9%, 87.6% and 13.9% indicated correctly the association between adhesions and age above 60 years, history of abdominal surgery and a history of Crohn’s disease respectively.

### Informed consent

**Fig 1:**
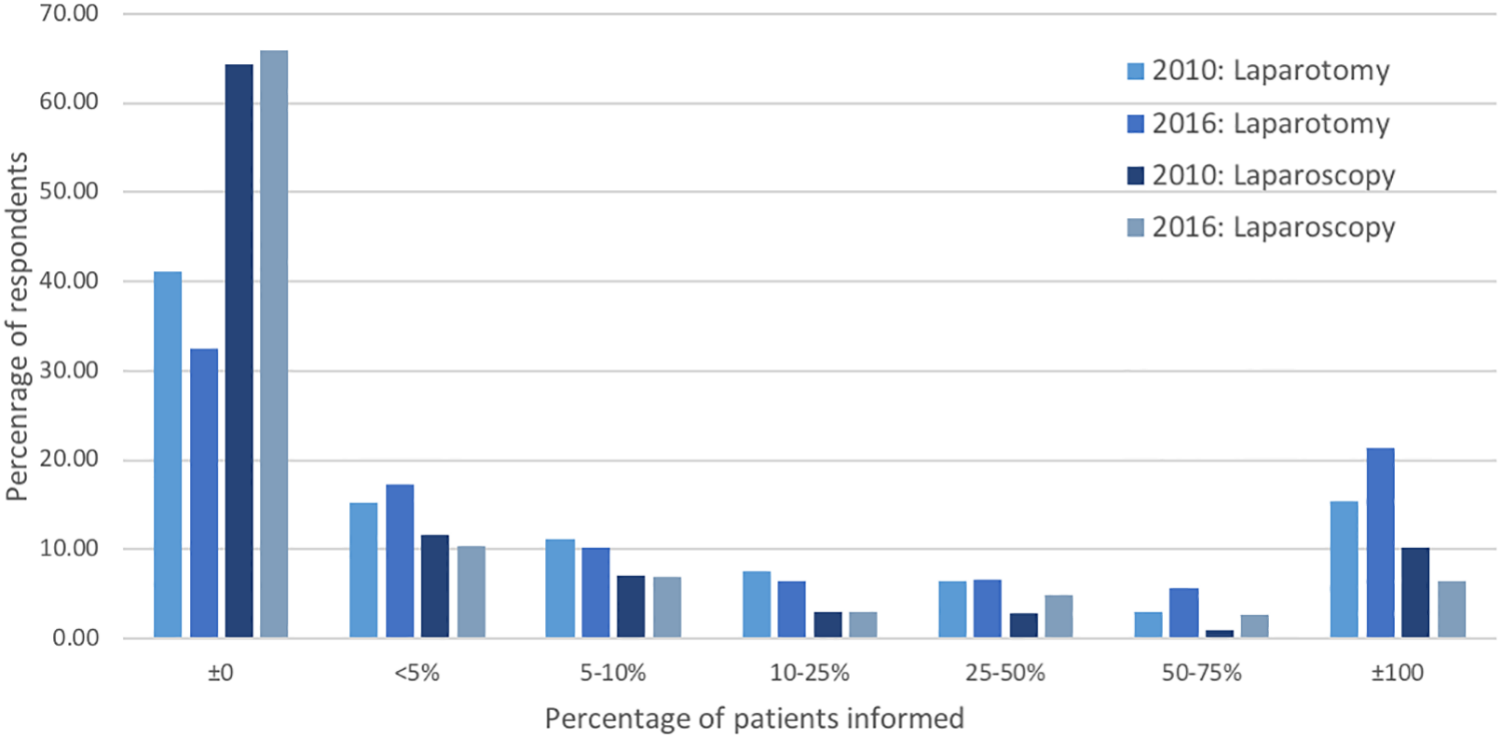
Informed consent. Percentage of patients informed regarding adhesion related complications after laparoscopy and laparotomy in 2010 and 2016.

Regarding laparotomy, 32.5% of the respondents reported that they almost never mention adhesions and related morbidity during the informed consent procedure, while 21.3% always report this complication risk (Fig 1). In the past six years this has increased, in the 2010 survey 41.1% never mentioned and 15.4% always mentioned adhesions and its morbidity preoperatively. This difference was not significant after correcting for multiple testing. Furthermore, 65.9% did not inform patients about the risk on adhesions before a laparoscopic procedure, which was comparable with the response from the 2010 survey. No differences between the subgroups were detected.

### Adhesion prevention and anti-adhesive barriers

Adhesion prevention for specific indications is believed to be more effective than adhesion prevention for all abdominal surgery (*p* < 0.001). Furthermore, gastrointestinal and general surgeons believed more strongly in the effectiveness of using meticulous surgical techniques than other groups (83.8%, *p* < 0.001). Residents believed the least in adhesion reduction by the use of such surgical techniques (residents 62.8% vs other 82%; *p* < 0.001). Furthermore, in the most recent survey more surgeons believe that laparoscopic surgery and the use of meticulous surgical techniques reduce adhesion formation (*p* <0.001 for both outcomes).

Of the respondents, 29.8% stated not to believe in anti-adhesive products with no differences between types of surgeons or residents. But interestingly, significantly more respondents indicated to like to apply adhesion prevention in all abdominal operation in 2016 compared to 2010 (45.8% vs 33.6% respectively, *p* = 0.001).

## Discussion

Although adhesions have a significant impact on patients’ quality of life and health care costs [4, 12], Dutch surgeons still underestimate the problem and there appears to be no improvement compared to the results of the similar survey conducted in 2010. The two most striking underestimates are the long-term morbidity and the need for fertility treatment as a consequence of intra-abdominal adhesions.

Even though almost 90% of Dutch surgeons consider adhesions as a clinical problem, only a quarter of all respondents inform patients regularly about the risk of adhesion related morbidity after laparotomy. In the case of laparoscopic interventions, this is even less. A striking discrepancy that was also concluded in the 2010 survey and also shows a meager improvement over a six-year time-period.

The apparent underestimates and poor involvement in the informed consent process could be related due to the fact that surgeons are not directly confronted with adhesions as a complication of operations performed by themselves.

The overall knowledge score of respondents in 2016 was approximately the same as in 2010, leading to the conclusion that adhesion awareness has not significantly changed over the last six years.

This survey showed that the awareness and knowledge of Dutch surgeons concerning complications related to adhesiolysis increased between 2010 and 2016. A bowel defect is seen in 40% of the surgical procedures, when adhesiolysis takes over an hour [3]. This is an important morbidity of intra-abdominal adhesions.

Unfortunately, adhesion formation can affect patients lifelong, considering the 30% readmission rate within 10 years postoperatively [4]. Although discussion regarding the true readmission rate is ongoing. A population based study, published after the conduction of the survey, showed a slightly lower readmission rate after 5 years (4.5%)[13] after open surgery. Others report a readmission rate of 6% within 4 years. But more interestingly is the readmission rate after laparoscopic surgery, which is higher than expected (3.0%)[13]. So, the positive effect of laparoscopic surgery in comparison to open surgery on adhesion formation seems to be overestimated.

Intra-abdominal adhesion research has been held back by the absence of a sound non-invasive diagnostic. Laparoscopy is the only definitive diagnostic, which is invasive, causes more adhesions and exposes the patient to the risk of iatrogenic bowel injury. The visceral slide, which is the normal longitudinal movement of the intra-abdominal viscera caused by respiratory excursion of the diaphragm, can be used for non-invasive diagnostic purposes. Transabdominal ultrasonography (TAU) and cine magnetic resonance (cine MRI) can detect abnormalities of the visceral slide that might be caused by intra-abdominal adhesions with an accuracy of 70% to 95%. TAU is limited in some patients due to intestinal gas or obesity and requires an extensive examination, which obstacles are overcome by cine MRI. However, prospective trials involving these non-invasive techniques are awaited [14].

Next, adhesions often cause abdominal pain and are associated with female infertility [3]. Taken this all into account, adhesions have a major impact on patient’s quality of life and moreover that they have a significant effect on health care costs. The use of anti-adhesive agents are shown to be cost-effective [12]. Surprisingly, the opinion of respondents regarding the effect of anti-adhesive agents did not change over the past six years. Although there is sufficient evidence that hyaluronate carboxymethylcellulose and regenerated cellulose reduce adhesion formation; the indication is a point of discussion. Heterogeneity, lack of long-term follow-up and a high variability in the quality of studies are reasons for reserved conclusions and thus more research is needed focusing on patient outcomes[15]. The lack of consensus is persistent in the last six years and no guidelines were drafted to aid the surgeon in clinical practice, in contrast to the gynecologists who published the 2012 European field guideline [16].

In the period between 2010 and 2016 an extensive review on the burden of adhesions and a meta-analysis regarding anti-adhesive products were published in high impact journals among others. Multiple conferences and meetings were organized to address the issue, but overall adhesion awareness did not increase. The factors responsible are subject to speculation, but the underestimation is most profound regarding problems involving adhesions that do not immediately return to the outpatient department of the surgeon. Female infertility is treated by the gynecologist, chronic abdominal complaints by the gastro-enterologist and complications occurring ten years from now are maybe considered not to be of the highest priority. Hopefully, with the development of new non-invasive diagnostic tools, such as the cine MRI, cause and effect will be linked more directly. The discrepancy between the effort in raising awareness and the actual effect on adhesion awareness is alarming. It shows that providing scientific information is not enough to change clinical practice and it calls for consensus in the form of guidelines, which are broadly carried by clinicians in the field.

Among the limitations of this study is the response rate, although comparable with the survey conducted in 2010 [7, 17]. A risk of selection bias is present. Surgeons with affinity for the burden of adhesions are more likely to respond, thus the results of this survey can be an underestimation of the problem. This survey was only distributed among Dutch surgeons, but worldwide extrapolation seems permissible.

Lastly, the surveys in 2010 and 2016 were filled in anonymously and for the statistical analysis considered as two independent samples of the same population, instead of pairing the surveys of participants.

In conclusion, adhesion awareness in the Netherlands did not increase between 2010 and 2016 despite all the efforts that were made during symposia, congresses, published meta-analysis and even newspaper articles. The awareness among surgeons regarding adhesiolysis related complications, as a significant morbidity caused by adhesions, has increased between 2010 and 2016. The disappointing effect on the adhesion awareness calls for a different approach. Courses should be developed and organized on a regular base. Furthermore, guidelines for preventing and treating adhesions are urgently needed.

## Supporting information

S1 Text Complete adhesion awareness survey 2016: The English version. (DOCX)
