# Supplementary material for: Adhesion awareness in 2016: An update of the national survey of surgeons
Source: PLoS One. 2018 Aug 17;13(8):e0202418. doi: 10.1371/journal.pone.0202418 (PMC6097683; doi:10.1371/journal.pone.0202418)
Supplement: S1 Text — (DOCX) [file pone.0202418.s001.docx]

**The National Adhesion Awareness Survey**

1. I participated in the Adhesion awareness survey 5 years ago:
   1. Yes
   2. No
2. You are... (multiple options possible)
   1. In training to become a surgeon
   2. General surgeon
   3. Oncologic surgeon
   4. Vascular surgeon
   5. Gastrointestinal surgeon
   6. Paediatric surgeon
   7. Trauma surgeon
3. How many years of work experience do you have as a surgeon or surgeon in training?
4. You currently work at a:
   1. Non-academic hospital.
   2. Academic hospital.
5. You currently work:
   1. Full-time.
   2. Part-time.
6. You are:
   1. Male.
   2. Female.
7. What is your opinion regarding intra-abdominal adhesions?
   1. Adhesions are clinically irrelevant.
      1. I strongly disagree.
      2. I disagree.
      3. I am Neutral.
      4. I agree.
      5. I strongly agree.
   2. Adhesions have more positive effects than negative.
      1. I strongly disagree.
      2. I disagree.
      3. I am Neutral.
      4. I agree.
      5. I strongly agree.
8. What is your opinion regarding adhesiolysis?
   1. Adhesiolysis because of diffuse abdominal complaints is pointless.
      1. I strongly disagree.
      2. I disagree.
      3. I am Neutral.
      4. I agree.
      5. I strongly agree.
   2. Adhesiolysis because of focal abdominal complaints is pointless.
      1. I strongly disagree.
      2. I disagree.
      3. I am Neutral.
      4. I agree.
      5. I strongly agree.
   3. Preferably, only specialised gastrointestinal surgeons should perform adhesiolysis.
      1. I strongly disagree.
      2. I disagree.
      3. I am Neutral.
      4. I agree.
      5. I strongly agree.
9. In how many patients with a small bowel obstruction are postoperative adhesions the cause?
   1. <25%
   2. 25 – 50%
   3. 50 -75%
   4. >75%
10. How many patients are readmitted 5 years after a laparotomy because of morbidity directly related to adhesions?
    1. 2%
    2. 5%
    3. 10%
    4. 20%
11. How many patients are readmitted 10 years after a laparotomy because of morbidity probably related to adhesions?
    1. 5%
    2. 10%
    3. 20%
    4. 30%
12. The complexity of an operation is measured by the time needed for adhesiolysis, because there is a relation with the risk of iatrogenic bowel injury.

    In how many operations, after one hour of adhesiolysis, is an iatrogenic bowel injury caused?
    1. <25%
    2. 25 – 50%
    3. 50 -75%
    4. >75%
13. The risk of adhesion formation is the highest in which surgical procedure?
    1. Partial small bowel resection
    2. Appendectomy
    3. Total (procto)colectomy
    4. Rectum resection
    5. Cholecystectomy
14. How many women undergo fertility treatment after abdominal surgery?
    1. <10%
    2. 10 – 20%
    3. 20 – 30%
    4. >30%
15. It is proven that some patient specific factors influence the formation of adhesions.
    1. age >60 year causes:
       1. Less adhesions.
       2. No effect.
       3. More adhesions.
    2. Previous operations causes:
       1. Less adhesions.
       2. No effect.
       3. More adhesions.
    3. M. Crohn causes:
       1. Less adhesions
       2. No effect
       3. More adhesions
    4. The use of prednisone causes:
       1. Less adhesions.
       2. No effect.
       3. More adhesions.
16. How many patients do you inform regarding adhesions or adhesion related complaints as a possible complication of a laparotomy?
    1. Almost none
    2. <5%
    3. 5 – 10%
    4. 10 – 25%
    5. 25 – 50%
    6. 50 – 75%
    7. Almost every patient
17. How many patients do you inform regarding adhesions or adhesion related complaints as a possible complication of a laparoscopic procedure?
    1. Almost none
    2. <5%
    3. 5 – 10%
    4. 10 – 25%
    5. 25 – 50%
    6. 50 – 75%
    7. Almost every patient
18. What is your opinion regarding adhesion prevention?
    1. You do not believe in adhesion prevention.
       1. I strongly disagree.
       2. I disagree.
       3. I am Neutral.
       4. I agree.
       5. I strongly agree.
       6. N/A
    2. You would like to apply adhesion prevention in all abdominal surgery.
       1. I strongly disagree.
       2. I disagree.
       3. I am Neutral.
       4. I agree.
       5. I strongly agree.
       6. N/A
    3. You would like to apply adhesion prevention only with specific indications.
       1. I strongly disagree.
       2. I disagree.
       3. I am Neutral.
       4. I agree.
       5. I strongly agree.
       6. N/A
    4. You are inclined to apply more adhesion prevention than five years ago.
       1. I strongly disagree.
       2. I disagree.
       3. I am Neutral.
       4. I agree.
       5. I strongly agree.
       6. N/A
19. What has influenced your opinion regarding adhesion prevention most in the past five years?
    1. Tuition/conference
    2. Publication in a medical journal
    3. Interaction with colleagues
    4. Your own experience with adhesions
    5. N/A
20. What is your opinion regarding the following statements?
    1. Laparoscopic surgery causes less adhesions than open surgery.
       1. I strongly disagree.
       2. I disagree.
       3. I am Neutral.
       4. I agree.
       5. I strongly agree.
    2. ... and you apply it as much as possible for that reason.
       1. I strongly disagree.
       2. I disagree.
       3. I am Neutral.
       4. I agree.
       5. I strongly agree.
    3. A meticulous surgical technique (tissue handling, avoiding the use of gauzes in the abdomen, etc.) causes less adhesions.
       1. I strongly disagree.
       2. I disagree.
       3. I am Neutral.
       4. I agree.
       5. I strongly agree.
    4. ... and you apply it as much as possible for that reason.
       1. I strongly disagree.
       2. I disagree.
       3. I am Neutral.
       4. I agree.
       5. I strongly agree.
    5. Extraperitoneal placement of a mesh causes less adhesions than intraperitoneal mesh placement.
       1. I strongly disagree.
       2. I disagree.
       3. I am Neutral.
       4. I agree.
       5. I strongly agree.
    6. ... and you apply it as much as possible for that reason.
       1. I strongly disagree.
       2. I disagree.
       3. I am Neutral.
       4. I agree.
       5. I strongly agree.
    7. A coated mesh causes less adhesions than a mesh without coating when its placed intraperitoneal.
       1. I strongly disagree.
       2. I disagree.
       3. I am Neutral.
       4. I agree.
       5. I strongly agree.
    8. ... and you apply it as much as possible for that reason.
       1. I strongly disagree.
       2. I disagree.
       3. I am Neutral.
       4. I agree.
       5. I strongly agree.
    9. The use of electrosurgery causes less adhesions.
       1. I strongly disagree.
       2. I disagree.
       3. I am Neutral.
       4. I agree.
       5. I strongly agree.
    10. ... and you apply it as much as possible for that reason.
        1. I strongly disagree.
        2. I disagree.
        3. I am Neutral.
        4. I agree.
        5. I strongly agree.
    11. Reducing the intraperitoneal use of suture material results in less adhesions.
        1. I strongly disagree.
        2. I disagree.
        3. I am Neutral.
        4. I agree.
        5. I strongly agree.
    12. ... and you apply it as much as possible for that reason.
        1. I strongly disagree.
        2. I disagree.
        3. I am Neutral.
        4. I agree.
        5. I strongly agree.
21. What is your opinion regarding products for adhesion prevention?
    1. You do not believe in products for adhesion prevention.
       1. I strongly disagree.
       2. I disagree.
       3. I am Neutral.
       4. I agree.
       5. I strongly agree.
    2. It is unclear when to use products for adhesion prevention.
       1. I strongly disagree.
       2. I disagree.
       3. I am Neutral.
       4. I agree.
       5. I strongly agree.
    3. You rather use products for adhesions prevention that act locally than a product with effect throughout the whole abdomen.
       1. I strongly disagree.
       2. I disagree.
       3. I am Neutral.
       4. I agree.
       5. I strongly agree.
    4. You think that the cost of products for adhesion prevention are disproportional in comparison to the possible advantages.
       1. I strongly disagree.
       2. I disagree.
       3. I am Neutral.
       4. I agree.
       5. I strongly agree.
22. What factors are contributory to your trust in products for adhesion prevention?
23. What factors are contributory to your distrust in products for adhesion prevention?
24. The following products for adhesion prevention you have…
    1. Interceed
       1. never used it.
       2. never used it but would like to.
       3. used it in the last year.
       4. used it once.
    2. Seprafilm
       1. never used it.
       2. never used it, but would like to.
       3. used it in the last year.
       4. used it once.
    3. Adept
       1. never used it.
       2. never used it, but would like to.
       3. used it in the last year.
       4. used it once.
    4. Spraygel
       1. never used it.
       2. never used it, but would like to.
       3. used it in the last year.
       4. used it once.
    5. Hyalobarrier
       1. never used it.
       2. never used it, but would like to.
       3. used it in the last year.
       4. used it once.
    6. Ringers lactaat
       1. never used it.
       2. never used it, but would like to.
       3. used it in the last year.
       4. used it once.
    7. Intercoat
       1. never used it.
       2. never used it, but would like to.
       3. used it in the last year.
       4. used it once.
25. For what indications have you used products for adhesion prevention?
    1. Relaparotomy because of adhesion related problems.
       1. Never
       2. Rarely
       3. Regularly
       4. Often
       5. Very often
    2. Pancreas- and duodenum surgery.
       1. Never
       2. Rarely
       3. Regularly
       4. Often
       5. Very often
    3. Partial small bowel resection
       1. Never
       2. Rarely
       3. Regularly
       4. Often
       5. Very often
    4. Creating an ileostomy
       1. Never
       2. Rarely
       3. Regularly
       4. Often
       5. Very often
    5. Appendectomy
       1. Never
       2. Rarely
       3. Regularly
       4. Often
       5. Very often
    6. Subtotal colectomy
       1. Never
       2. Rarely
       3. Regularly
       4. Often
       5. Very often
    7. Panproctocolectomy
       1. Never
       2. Rarely
       3. Regularly
       4. Often
       5. Very often
    8. Laparoscopic colon surgery
       1. Never
       2. Rarely
       3. Regularly
       4. Often
       5. Very often
    9. Rectum procedure
       1. Never
       2. Rarely
       3. Regularly
       4. Often
       5. Very often
    10. Abdominal wall surgery
        1. Never
        2. Rarely
        3. Regularly
        4. Often
        5. Very often
    11. Aortic bifurcation prostheses
        1. Never
        2. Rarely
        3. Regularly
        4. Often
        5. Very often
26. How many times are products for adhesion prevention used by you and your colleagues in your hospital?
    1. % of all laparotomies:
    2. % of all laparoscopic procedures:
27. Do you think that this percentage has increased over the last five years?
    1. Yes
    2. No
    3. Can you explain your answer?
28. Do you have a last remark regarding adhesions?
29. Your email-address:
